# Faustovirus-Like Asfarvirus in Hematophagous Biting Midges and Their Vertebrate Hosts

**DOI:** 10.3389/fmicb.2015.01406

**Published:** 2015-12-16

**Authors:** Sarah Temmam, Sonia Monteil-Bouchard, Masse Sambou, Maxence Aubadie-Ladrix, Saïd Azza, Philippe Decloquement, Jacques Y. Bou Khalil, Jean-Pierre Baudoin, Priscilla Jardot, Catherine Robert, Bernard La Scola, Oleg Y. Mediannikov, Didier Raoult, Christelle Desnues

**Affiliations:** ^1^Unité de Recherche sur les Maladies Infectieuses et Tropicales Emergentes, UM63 Centre National de la Recherche Scientifique 7278 IRD 198 Institut National de la Santé et de la Recherche Médicale U1095, Aix-Marseille UniversitéMarseille, France; ^2^Unité de Recherche sur les Maladies Infectieuses et Tropicales Emergentes, UM63 Centre National de la Recherche Scientifique 7278 IRD 198 Institut National de la Santé et de la Recherche Médicale U1095, Aix-Marseille UniversitéDakar, Senegal; ^3^Fondation IHU Méditerranée Infection, Pôle des Maladies Infectieuses et Tropicales Clinique et Biologique, Fédération de Bactériologie-Hygiène-Virologie, Centre Hospitalo-Universitaire Timone, Méditerranée Infection, Assistance Publique – Hôpitaux de MarseilleMarseille, France

**Keywords:** biting midges, giant virus, faustovirus, bloodmeal host, environment

## Abstract

Faustovirus, a new *Asfarviridae*-related giant virus, was recently isolated in *Vermamoeba vermiformis*, a protist found in sewage water in various geographical locations and occasionally reported in human eye infection cases. As part of a global metagenomic analysis of viral communities existing in biting midges, we report here for the first time the identification and isolation of a Faustovirus-like virus in hematophagous arthropods and its detection in their animal hosts. The DNA virome analysis of three pools of *Culicoides* sp., engorged female *Culicoides imicola* and non-engorged male/female *C. imicola* biting midges collected in Senegal, revealed the presence of amoeba-infecting giant viruses and, among them, a majority of sequences related to Faustovirus. Phylogenetic analyses conducted on several structural genes of Faustovirus confirmed the clustering of the arthropod-borne Faustovirus with sewage-borne Faustoviruses, with a distinct geographical clustering of Senegalese Faustovirus strains. Transmission electron microscopy identified viral particles with morphologies and diameters which were compatible with Faustovirus. The presence of infectious arthropod-borne Faustovirus was finally confirmed by successful isolation on *V. vermiformis* amoeba. Global proteomic analysis of biting midges identified that arthropods' blood meal originating from cattle, rodents and humans. Further screening of cattle sera and rodent tissue resulted in prevalence of Faustovirus being estimated at 38% in rodents and 14% in cattle, suggesting a possible origin of Faustovirus presence in arthropods via the ingestion of contaminated blood meal. Viral loads were the highest in rodents' urine and kidney samples, suggesting a possible excretion of viral particles into the environment. Faustovirus DNA polymerase-related sequences were also detected in more than 9 and 11% of febrile patients and healthy Senegalese human sera, respectively. Our study thus, highlights the need to investigate the role of arthropods, wildlife, and domestic animals in the lifecycle of amoeba-infecting giant viruses and, in particular, the environmental cycle of Faustovirus.

## Introduction

Large double-stranded (ds)DNA viruses, also known as “giant viruses,” form a monophyletic group consisting of *Poxviridae, Iridoviridae, Ascoviridae, Phycodnaviridae, Asfarviridae, Mimiviridae*, and *Marseilleviridae* families and are classified under the proposed *Megavirales* order (Colson et al., 2012, 2013). More recently, discovery of *Pandoravirus* and *Pithovirus* genera has been reported (Philippe et al., 2013; Legendre et al., 2014).

Protozoans, and especially amoebas, have been largely used as tools to isolate and cultivate a wide variety of micro-organisms, due to their lack of receptor-dependent infection and the ability of some bacteria and viruses to resist phagocytosis and to multiply in these organisms (Greub and Raoult, 2004). So far, giant viruses have been isolated on amoebae from various environments all over the world, mostly from water samples (Pagnier et al., 2013). Recently Faustovirus, a new virus closely related to the *Asfarviridae* family, has been isolated on *Vermamoeba vermiformis* protists in sewage water in various geographical locations (Reteno et al., 2015). *Asfarviridae* are a family of dsDNA viruses consisting of a unique member: the African swine fever virus (ASFV), the only known dsDNA virus transmitted by hematophagous arthropods, i.e., ticks.

*Ceratopogonidae*, and especially the genus *Culicoides*, are well-known vectors of several parasites (Agbolade et al., 2006; Slama et al., 2014) and viruses (Mellor et al., 2000) infecting animals and human (i.e., Bluetongue virus, African Horse Sickness virus, Epizootic Hemorrhagic Disease virus, and Oropouche virus, the only known human virus transmitted by biting midges). In sub-Saharan countries such as Senegal, biting midges usually feed on livestock but also on humans. Larval stages of *Culicoides* sp. are found in semi-aquatic environments (Harrup et al., 2013), leading to possible contact with amoebae and their associated giant viruses.

In the present study, we report for the first time the detection, isolation, and environmental exploration of Faustovirus in adult *Culicoides* sp. biting midges.

## Materials and methods

### Sample collection and ethical statement

#### Arthropods

**Figure 1:**
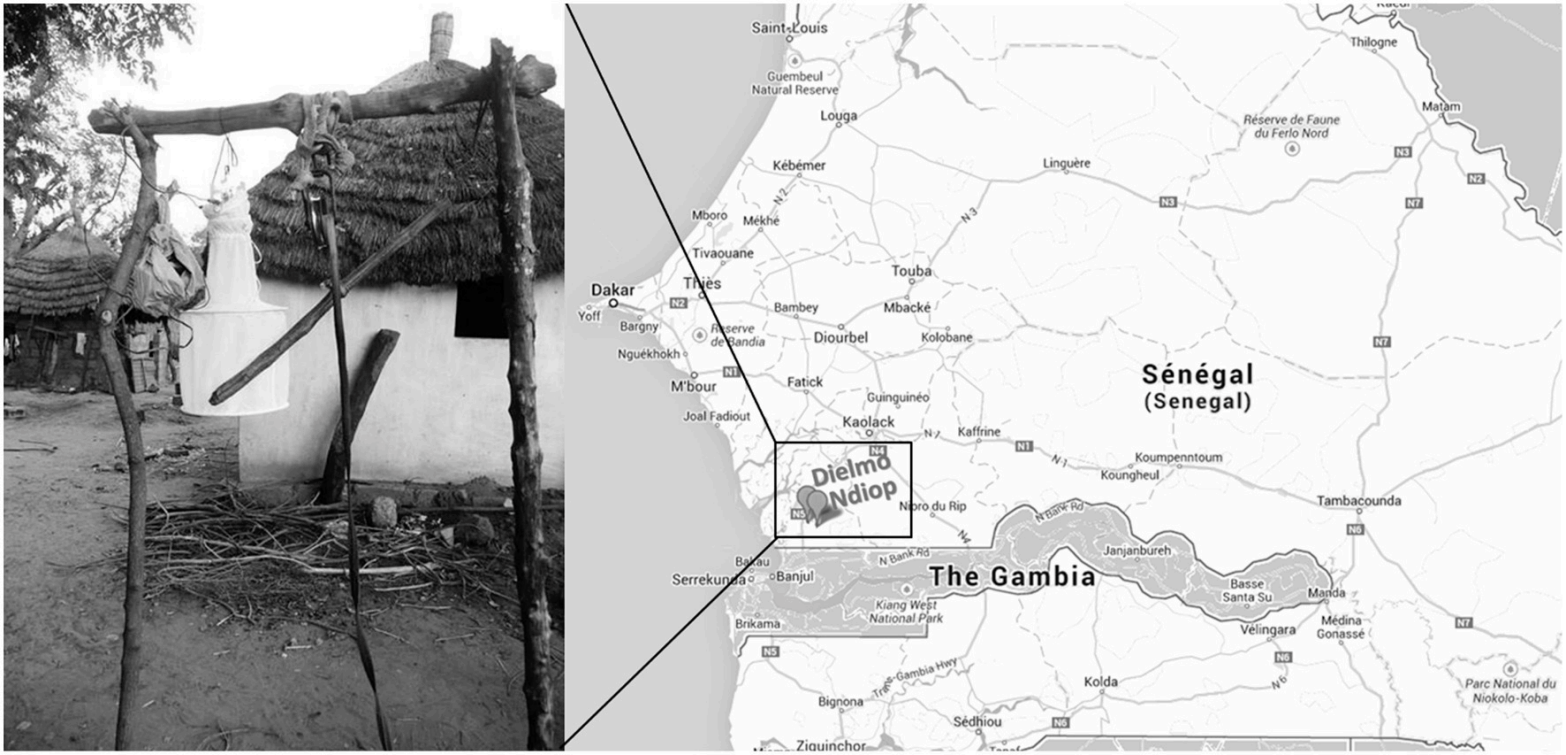
**Sampling sites**.

Biting midges were collected using a modified CDC light trap as previously described (Sambou et al., 2015), in the villages of Dielmo and Ndiop in the Sine-Saloum region of Senegal, in November 2013 (Figure 1). Traps were placed near places where cattle rested and were left overnight. Morphological identification of the arthropods was conducted the next morning, as previously described by Sambou et al. (2015). Three types of arthropod pools were created: the STE0043 pool consisted of more than 200 adult *Culicoides* sp., with no distinction between male and female, nor their gorged status; STE0044 and STE0045 pools consisted of 15 engorged female and 100 non-engorged male and female *Culicoides imicola*, respectively. Arthropods were immediately stored in liquid nitrogen directly in the field. All these pools were collected from the same concession in Dielmo during the same night.

Hard ticks collected from cattle were harvested and directly stored in liquid nitrogen in pools according to their animal origin.

#### Cattle

Cattle sera were collected from animals at the same location as the CDC light traps used to sample biting midges. These were also immediately stored in liquid nitrogen.

#### Rodents

Rodent trapping was conducted at the same place: traps were left open overnight, and small mammals were sacrificed the next morning by cervical dislocation, according to the guidelines for the handling of wild mammals (Sikes and Gannon, 2011). All animal procedures carried out in this study were approved by the IRD Local Ethics Committee. The spleen, lungs, kidney, liver, brain, bladder, intestine, and serum were collected from trapped animals and directly stored individually in liquid nitrogen. Species identification of trapped small mammals was conducted by sequencing the 18S rRNA, as previously described (Breitbart and Rohwer, 2005).

#### Water

Drinking water collected from wells in the two rural villages of Dielmo and Ndiop, and water collected from the Néma river in Dielmo were filtered through a 0.80-μm filter, followed by a 0.45-μm filter (Millipore, Molsheim, France). 10% (w/v) of PolyEthylenGlycol (PEG6000, Sigma Aldrich, Saint-Quentin Fallavier, France) and 300 mM NaCl (Sigma Aldrich, Saint-Quentin Fallavier, France) were then added to precipitate viral particles and were incubated overnight at +4°C. After centrifugation at 12,000 g for 20 min, the final pellet was resuspended in 2 mL of 0.02 μm-filtered PBS and stored in aliquots at −80°C.

#### Human

Human sera were collected through the Point-of-Care (POC) laboratory in Dielmo (Sokhna et al., 2013). One hundred and twelve sera from febrile people with no known etiology and 51 sera from healthy people were collected between November 2013 and June 2014. The National Ethics Committee of Senegal approved the most recent protocol, including the POC laboratory and activities, under the “Avis éthique et scientifique n°00081 du 04 juin 2012.”

### Sample processing

Fifty microliters of cattle sera, up to 20 μL of rodent urine and 100 μL of PEGylated water pellets were used to extract total nucleic acids using the High Pure Viral Nucleic Acids kit (Roche Diagnostics, Mannheim, Germany), according to the manufacturer's instructions.

Approximately 0.5 cm^3^ of rodent tissues and up to five hard ticks were crushed in pools with two 3-mm tungsten beads and a TissueLyser at 25 Hz for 2 min (Qiagen, Courtaboeuf, France) in 2 mL of sterile EMEM (Life Technologies, Saint Aubin, France). Two hundred microliters of clarified supernatant was then processed for nucleic acid extraction as for cattle sera.

Total nucleic acids from human sera were previously extracted within the POC laboratory in Dielmo. Briefly, 200 μL of human capillary blood was extracted using the Nucleospin Tissue kit (Macherey-Nagel, Hoerdt, France), according to the manufacturer's recommendations.

### Virome preparation

The three pools of arthropods were crushed with two 3-mm tungsten beads and a TissueLyser at 25 Hz for 2 min (Qiagen, Courtaboeuf, France). The clarified supernatant was subsequently used as a template for virome preparation.

DNA viromes were prepared to purify viral particles from their complex sample. Briefly, the clarified supernatant was filtered through a 0.45-μm filter (Millipore, Molsheim, France), and free nucleic acids were digested with a cocktail of nucleases, i.e., 20U of Turbo DNAse (Life Technologies, Saint Aubin, France), 25U of RNAse A (Roche Diagnostics, Meylan, France), 25U of Benzonase® (Merck Millipore, Molsheim, France) and 20U of Exonuclease I (New England Biolabs, Évry, France), as previously described (Temmam et al., 2015). Finally, the digested supernatant was purified onto a discontinuous 66–30% sucrose gradient and ultracentrifuged at 130,000 g for 2 h at +4°C on a MLS-50 rotor (Beckman-Coulter, Villepinte, France). The viral fraction was harvested at the interphase between the 66 and 30% sucrose layers.

DNA was extracted from the purified viral fraction using Trizol LS® reagent (Life Technologies, Saint Aubin, France), according to the manufacturer's recommendations, and was randomly amplified using a Genomiphi V3 kit (GE Healthcare, Vélizy-Villacoublay, France) in two independent reactions. Amplification products were pooled and purified with Agencourt AMPure Beads (Beckman-Coulter, Villepinte, France) according to the manufacturer's protocol, eluted to a final volume of 15 μL and sequenced using MiSeq Technology with the paired-end and barcode strategies according to a Nextera XT library kit in a 2 × 300 bp format (Illumina Inc., San Diego, USA).

### Bioinformatic analyses of viromes

Raw reads were imported in pairs into CLC Genomics Workbench 6.0.1 program (CLC Bio, Aarhus, Denmark) and trimmed according to their quality score, the presence of ambiguities, and their length (reads < 50 nt long were discarded). The pre-processed viral metagenomes are publicly available on the Metavir server (http://metavir-meb.univ-bpclermont.fr) under the project “Arthrovirome” project and on the MG-RAST server (http://metagenomics.anl.gov/) with the identifiers 4604224.3, 4604225.3, and 4604226.3 for the STE0043, STE0044, and STE0045 DNA viromes, respectively.

Cleaned paired reads were assembled into contigs with the CLC Genomics program using the following parameters: a word size of 20 nt, minimum contig length of 200 nt, mismatch cost of 2, insertion/deletion cost of 3, length fraction of 0.5, and similarity fraction of 0.8. Contigs and non-assembled reads were then compared to the NCBI nucleotide database using the BlastN algorithm, with a minimum coverage of 50%, minimum identity of 50%, and an *E*-value < 10^−5^. Sequences with no significant hits according to the criteria described above were classified as “unknown.” Contigs were then compared to the *Megavirales* database (Verneau et al. [METADIG: an automated pipeline to search for giant virus-related sequences in metagenomes. *In revision*]) using the BlastX program with a minimum coverage of 50%, minimum identity of 50%, and an *E*-value < 10^−5^. To confirm the specificity of the BlastX result, contigs were finally compared to the NCBI RefSeq viral database and to the whole NCBI database under the same criteria. The taxonomic assignment of contigs was conducted by selecting the best BlastX score result between the three Blasts run for each contig.

### Phylogenetic analyses

Contigs matching with amoeba-infecting giant viruses were extracted and translated using the FragGeneScan tool (Rho et al., 2010), according to the “short” and “complete” parameters. Predicted Open Reading Frames (ORF) were then compared to the *Megavirales* database using the BlastP program to identify *Megavirales* core genes. Phylogenetic analyses were performed on the amino-acid sequences of the RNA diphosphate reductase large sub-unit and the nucleotide sequence of the sub-unit common to RNA polymerase I–II–III, the DNA topoisomerase and the putative helicase C962R of Faustovirus.

Amino-acid and nucleotide sequences were retrieved from the GenBank database and aligned with the MUSCLE aligner (Edgar, 2004) implemented through MEGA6 (Tamura et al., 2013). The DNA/amino-acid substitutions model that best fitted the data were performed on MEGA6 (Tamura et al., 2013) and were considered for all phylogenetic analyses. We selected the best substitution model using the corrected Akaike information criterion. Phylogenetic trees were constructed by Maximum Likelihood (ML) implemented through the MEGA6 package software, according to the selected substitution model. Nodal support was evaluated by 1000 bootstrap replicates.

### Detection of Faustovirus in animal, human, and environmental samples

Quantitative SYBR Green real-time PCR targeting the DNA polymerase of Faustovirus was performed with the Quantitect SYBR Green qPCR kit (Qiagen, Courtaboeuf, France), according to the manufacturer's recommendations, except that 400 nM of forward (5′-CAAAGGCTATTGAGGCGATTTG-3′) and reverse (5′-ATGATTGTGCTGCTAGGATACC-3′) primers were used and mixed with 5 μl of DNA. Annealing temperature was defined as 58°C.

A standard curve was generated after extraction of serial dilutions of a flow cytometer-quantified Faustovirus. Briefly, the quantification was realized using a suspension of concentration-calibrated Cytocount™ fluorescent beads (Dako, Les Ulis, France) and the following formula: (number of counted particles / number of counted beads) × bead concentration (i.e., 1100 beads/μL), as previously reported (Khan et al., 2010). The resulting count was expressed in Virus-Like Particles (VLP) per mL. Dilutions of the quantified virus were performed, and nucleic acids were extracted from each dilution and further used as template for the qPCR standard curve.

Primers targeting Faustovirus RNA polymerase and DNA topoisomerase were designed according to the metagenomes sequences: Fausto_RNApol_F (5′-TACGTCAAGCAGTAGCCA ACG-3′), Fausto_RNApol_R (5′-CTACTTGCCGCACAA CAGCC-3′), Fausto_DNAtopo_F (5′-CCAGCACCATATGACACG CG-3′) and Fausto_DNAtopo_R (5′-AATGTATGCGTTCGATTCGCC-3′). PCR targeting Faustovirus RNA polymerase, DNA topoisomerase and capsid (Reteno et al., 2015) were performed using the Hot Star Taq DNA polymerase (Qiagen, Courtaboeuf, France). Annealing temperatures were 57°C, 57°C, and 58°C, respectively.

All PCR products were further analyzed on a 2% agarose gel, and bands of the expected size were extracted from the gel, purified using the QIAex Gel Extraction kit (Qiagen, Courtaboeuf, France) and sequenced with a Big Dye Terminator v1.1 Cycle Sequencing Kit (Life Technologies, Saint Aubin, France) and an ABI 3130 Genetic Analyzer (Life Technologies, Saint Aubin, France).

### Total protein extraction, western blot, and global proteomic analyses

Approximately 50 mg of arthropods from the STE0043 sample were crushed in 300 μL of lysis buffer (Tris-HCl 40 mM pH 7.5, SDS 2% (w/v), DTT 60 mM) with two 3-mm tungsten beads and a TissueLyser at 25 Hz for 2 min (Qiagen, Courtaboeuf, France) before heating at 95°C for 5 min. Proteins from the clarified supernatant were precipitated using the PlusOne 2-D Clean-Up Kit (GE Healthcare, Vélizy-Villacoublay, France). The final pellet was resuspended in 200 μL of solubilization buffer (Urea 8M, Thiourea 2M, 100 mM NaCl, 25 mM Tris, pH 8.2) and dialyzed twice using Slide-ALyzer Dialysis Cassettes 2K MWCO (Pierce Biotechnology, Rockford, USA) against 1 L of 50 mM ammonium bicarbonate pH 7.4, Urea 1M (7 h and overnight). Dialyzed fractions were collected and proteins were quantified by Bradford assay using Coomassie (Biorad, Marnes-la-Coquette, France). The dialyzed fraction was subsequently used as a template for global proteomics and western blot analyses.

Two hundred micrograms of soluble proteins were fractionated on a 12% polyacrylamide gel electrophoresis then revealed by silver staining. Additionally, resolved proteins were transferred onto a nitrocellulose membrane (Trans-blot Transfer Medium, Biorad, Hercules, CA, USA) at 100 V for 1 h using a semi-dry transfer unit (Hoefer TE 77, GE Healthcare, Vélizy-Villacoublay, France). Membranes were then blocked in PBS supplemented with 0.3% Tween-20 and 5% non-fat dried milk (PBS-Tween-Milk) for 90 min, and incubated with mouse polyclonal anti-Faustovirus antibodies (1:1000). The immunoreactive bands were detected using a peroxidase-conjugated goat anti-mouse immunoglobulin (GE Healthcare, Vélizy-Villacoublay, France) diluted to 1:5000 in the blocking buffer for 1 h at room temperature. Three fifteen minutes washes were applied between each step. Immunostained bands were visualized with the chemiluminescence-based kit, as described by the manufacturer (GE Healthcare, Vélizy-Villacoublay, France). The resulting signal was captured by a Fusion FX7 imaging system (Vilber Lourmat, France).

Six pieces corresponding to immunoreactive bands were excised from silver stained gel and subject to mass spectrometry (MS) analysis. Briefly, after several washes, the proteins extracted from the gel were reduced, alkylated and digested with trypsin, as described above. Tryptic peptides were extracted with acetonitrile 100%; the extraction solution was collected and incubated at 45°C to evaporate the acetonitrile and to concentrate it prior to MS analysis. An additional global proteomic analysis was conducted. Briefly, 200 μg of total soluble proteins were reduced and alkylated with iodoacetamide. Protein digestion was performed by adding 8 μg of sequencing-grade trypsin solution (Promega, Charbonnières, France) to alkylated proteins and incubated overnight at 37°C. The digested sample was then desalted using Pierce Detergent Removal Spin Columns (Thermo Fisher Scientific, Illkirch, France) and analyzed by mass spectrometry, as described hereafter.

A NanoAcquity UPLC System (Waters, Saint-Quentin En Yvelines, France) was coupled with a Synapt-G2 Si HDMS with Traveling-Wave-Ion-Mobility Mass Spectrometry instrument (TWIM-MS; Waters, Saint-Quentin En Yvelines, France). Chromatographic separation was performed on an NanoAcquity UPLC BEH130 C18 column (1.7 μm, 100 μm × 100 mm; Waters, Saint-Quentin En Yvelines, France) preceded by a Symmetry C18 trapping column (5 μm, 180 μm × 20 mm); both were placed in a 40°C oven. The injection volume was set to 2 μL for the digested soluble proteins (200 ng/μL) and 4 μL for gel-extracted proteins. The mobile phase consisted of water (A) and acetonitrile (B) both in 0.1% formic acid. Samples were trapped over 3 min with 99.9% A and 0.1% B. The separation gradient was as follows: 0–100 min, linear from 95% A, 5% B, to 60% A, and 40% B; 100–107 min. Mass spectrometry experiments were performed in positive ion mode and in resolution mode. The settings of the instruments were automatically optimized to obtain the best resolution. The ion source parameters were capillary voltage 3 kV, sampling cone voltage 40 V, ion source temperature 90°C and cone gas flow 50 L/h. Transfer collision low energy was set to 5 V while trap collision low energy was set to 4 V. The high energy ramp was applied from 4 to 5 V for the trap collision and from 19 to 45 V for the transfer collision, enabling fragmentation of the ions after the ion mobility cell and before the time-of-flight (TOF) MS. The instrument was previously calibrated in the mass range of 50–2000 Da using GFP fragments (0.2 pmol/μL). Data were processed using ProteinLynx Global Server version 3.0.1 (Waters, Saint-Quentin En Yvelines, France). Processing parameters were 250 counts for the low energy threshold, 100 counts for the elevated energy threshold and 750 counts for the intensity threshold.

Databases used to compare spectra combined data from Mammalia (2015/Feb/09, Swissprot, 66,370 sequences), Dipteria (2015/Feb/09, Swissprot, 6607 sequences), and giant viruses (2015/Feb/06, TrEMBL and not published giant viruses sequences, 14,866 sequences). An additional database was generated using predicted ORFs generated following the FragGeneScan analysis of giant viral contigs of the three metagenomes. A cut-off was used to remove the matches with only one and two peptides and the option of Merge Data was used with the six gel pieces.

### Transmission electron microscopy (TEM)

Approximately 50 mg of arthropods from the STE0043 sample were washed in 70% ethanol, as previously described (Slimani et al., 2013) and crushed in 2 mL of sterile EMEM medium (Life Technologies, Saint Aubin, France) using two 3-mm tungsten beads and a TissueLyser at 25 Hz for 2 min (Qiagen, Courtaboeuf, France). The supernatant was harvested after a low speed clarification and subsequently filtered through a 0.8-μm filter (Millipore, Molsheim, France). The resulting supernatant was purified onto a discontinuous 66–30% sucrose gradient and ultracentrifuged at 130,000 g for 2 h at +4°C, as described above.

The viral fraction was harvested at the interphase between the 66 and 30% sucrose layers and fixed for 1 h at +4°C with 2% final glutaraldehyde. The fixed viral fraction was then diluted to a final volume of 4 mL in PBS, and adsorbed directly onto formvar carbon films on 400 mesh nickel grids (FCF400-Ni, EMS) by ultracentrifugation at 130,000 g for 1 h at +4°C, as previously described (Sime-Ngando et al., 1996). Grids were stained for 10 s with 1% molybdate solution in filtered water at room temperature. Electron micrographs were obtained on a Tecnai G2 transmission electron microscope (FEI) operated at 200 keV equipped with a 4096 × 4096 pixel resolution Eagle camera (FEI).

### Isolation of viruses on amoebae

Approximately 50 mg of arthropods from the STE0043 sample were washed in 70% ethanol, as previously described (Slimani et al., 2013), then with sterile Page's amoebal saline (PAS) solution, and finally crushed in 3 mL of PAS buffer.

*V. vermiformis* (CDC19 strain) amoebae were used to isolate giant viruses from arthropods, as reported by Pagnier et al. (2013), except that Vancomycin 10 μg/mL, Ciprofloxacine 20 μg/mL, Imipenem/cilastatine 10 μg/mL, Doxycycline 20 μg/Ml, and Voriconazole 20 μg/mL were added to the amoebal suspensions to prevent bacterial and fungal contamination. Briefly, amoebae were cultivated in peptone-yeast extract-glucose (PYG) medium and sub-cultured every 2 days. 5 × 10^5^ amoebae/mL were concomitantly plated in a 12-well microplate with 100 μL of the sample suspension and incubated at 30°C for 3 days. At Day 3 post-infection, the primo-culture was sub-cultured onto a fresh amoebal microplate suspension under the same conditions. The primary cultures and sub-cultures were screened daily for a cytopathogenic effect (CPE) using an inverted microscope and if CPEs were observed, fresh amoeba cells were cytospinned with 100 μL of viral supernatant and further stained with Gimenez and Gram stains, followed by additional Hemacolor staining (Merck, Darmstadt, Germany).

Additionally, 100 μL of positive CPE supernatant was used to extract DNA using the phenol chloroform isoamyl alcohol extraction procedure (Life Technologies, Saint Aubin, France) according to the manufacturer's protocol, and PCRs targeting Faustovirus were conducted, as described above.

## Results

### Presence and diversity of sequences related to giant viruses in the virome of biting midges

The DNA virome of the STE0043, STE0044, and STE0045 samples were sequenced using Illumina MiSeq technology. After trimming, the total number of reads was 1,517,965, 2,163,868, and 2,265,552 reads, respectively (Table 1), with 46.29, 76.01, and 48.50% of sequences having homologies after BlastN against GenBank nt database, respectively. Eukaryotic sequences represented more than 70% of the total assigned reads in the STE0043, STE0044, and STE0045 metagenomes, mainly identified as human and arthropod reads (Table 1).

**Table 1:** **Sequencing data of the virome datasets**.

	**STE0043 *Culicoides* sp**.	**STE0044*C. imicola* ♀engorged**	**STE0045 *C. imicola*♂♀non-engorged**
Raw reads	1,520,202	2,173,228	2,267,752
Cleaned reads	1,517,965	2,163,868	2,265,552
Contigs	19,771	29,995	28,309
Singletons	85,185	134,230	122,805
Average contig length	615 bp	587 bp	581 bp
Total assigned reads:	702,730	1,644,792	1,098,862
eukaryote	576,221	1,457,350	781,498
prokaryote (bacteria + archaea)	122,075	164,982	303,928
virus	4434	22,460	13,436
Total giant viruses:	3465	20,745	4684
Faustovirus	3146	8383	3490
*Mimiviridae*	317	12,362	1164
Non-classified giant viruses	2	0	30
Other viruses:	969	1715	8752
*Nudiviridae*	307	0	6237
*Poxviridae*	182	42	34
*Siphoviridae*	182	503	808
Non-classified phages	156	277	240
*Myoviridae*	60	554	785
*Papillomaviridae*	41	100	2
*Iridoviridae*	29	0	0
*Podoviridae*	4	45	29
*Phycodnaviridae*	2	90	133
*Polydnaviridae*	2	0	0
*Retroviridae*	2	92	16
Non-classified plant viruses	2	0	0
*Ascoviridae*	0	12	2
*Herpesviridae*	0	0	315
*Inoviridae*	0	0	149
*Iflaviridae*	0	0	2

Among the total assigned reads, 4434 (STE0043), 22,460 (STE0044), and 13,434 (STE0045) sequences were related to viruses (Table 1). Most viral sequences were identified as double-stranded DNA viruses, and single-stranded DNA viral sequences were only detected in STE0045 non-engorged male and female *C. imicola* virome. Sequences related to amoeba-infecting giant viruses from the *Mimiviridae* family, Faustovirus and the non-taxonomically classified Pandoravirus represented the majority of viral reads, with more than 78, 92, and 34% of total viral reads, respectively, for STE0043, STE0044, and STE0045 viromes. The most represented virus was Faustovirus, with more than 90, 40, and 74% of total giant viral reads, respectively (Table 1).

The presence of Faustovirus in each sample was confirmed by PCR specifically targeting the DNA polymerase, the viral capsid, the RNA polymerase and the DNA topoisomerase genes of the virus. PCR amplification products were obtained for the three metagenomes (see Supplemental Figure 1 for the capsid amplification) and Faustovirus amplifications were confirmed by sequencing.

The presence of amoeba-infecting giant viral sequences was searched on previously published arthropods metagenomes available in public databases (Table 2). Sequences from both hematophagous (mosquitoes, hard ticks, and body lice) and non-hematophagous (termites and whiteflies) arthropods were assembled into contigs and compared to an in-house giant viral database. Mosquito microbiomes showed the presence of *Mimiviridae* and *Pandoraviridae*-related contigs in all of the five studies, although these came from arthropods sampled at different time points in different locations. Hard ticks and experimentally-infected body lice metagenomics revealed also the presence of *Mimiviridae*-related contigs. In contrast, termites and whiteflies metagenomic datasets did not present any amoeba-infecting giant viral contigs. No Faustovirus-related sequences were retrieved in metagenomes, either from hematophagous or non-hematophagous arthropods.

**Table 2:** **Search for the presence of amoeba-infecting giant viral sequences in the metagenomes of other arthropods**.

**Arthropods**	**Nb of total reads**	**Nb of contigs**	**Sequencing method**	**Type of metagenome**	**Amoeba-infecting giant viral contig (nb)**	**References**
Mosquitoes	1,575,043	1964	Roche 454 FLX	RNA shotgun	*Mimiviridae* (1)	Bishop-Lilly et al., 2010
	1,961,290	16,321	Roche 454 GS20	DNA shotgun	*Mimiviridae* (1) *Pandoraviridae* (1)	Dinsdale et al., 2008
	26,403,284	89,744	Illumina GA II	RNA shotgun	*Mimiviridae* (8) *Pandoraviridae* (10)	Chandler et al., 2014
	217,330,434	311,750	Illumina HiSeq 2000	RNA shotgun	*Mimiviridae* (13) *Pandoraviridae* (19)	Chandler et al., 2015
	1,576,489	15,666	Roche 454 GS20	DNA shotgun	*Mimiviridae* (1) *Pandoraviridae* (2)	Ng et al., 2011
Body lice	4,403,873	1733	Illumina MiSeq	RNA shotgun	*Mimiviridae* (5)	Temmam et al., 2015
Whiteflies	1,427,809	193	Illumina GA II	RNA shotgun	0	Rosario et al., 2014
Termites	–	57,641	Sanger	DNA shotgun	0	Warnecke et al., 2007
Hard ticks	350,329	31,881	Roche 454 FLX	DNA shotgun	*Mimiviridae* (1)	Nakao et al., 2013

### Phylogenetic analyses of the Faustovirus-like virus identified in the virome of biting midges

Contigs matching with Faustovirus sequences were extracted and translated. Results of the predicted ORFs are presented in Table 3. Phylogenetic reconstructions were performed on several conserved genes: the RNA diphosphate reductase large sub-unit and the sub-unit common to RNA polymerase I–II–III that were found in the three biting midges' metagenomes, the DNA topoisomerase only detected on the STE0043 *Culicoides* sp. virome and the putative helicase C962R, both present in the STE0044 *C. imicola* engorged female and the STE0045 *C. imicola* non-engorged male/female viromes.

**Table 3:** **Predicted ORFs for Faustovirus detected in the three metagenomes**.

	**STE0043**		**STE0044**		**STE0045**	
	**Short**	**Complete**	**Short**	**Complete**	**Short**	**Complete**
Total contigs/reads	79/3146		137/8383		114/3490	
TOTAL ORFs	93	87	148	145	127	125
Hypothetical protein	83	70	125	122	105	103
62 kDa polyprotein	–	–	1	1	1	1
Ankyrin containing protein	–	–	1	1	1	1
Bacterial MORN repeat-containing protein	–	2	2	2	–	–
BTB/POZ domain-containing protein	–	–	1	1	2	2
BTB/POZ domain-containing protein 9	1	–	1	1	–	–
Deoxyuridine 5′-triphosphate nucleotidohydrolase	–	–	2	2	–	–
DNA topoisomerase small subunit	–	1	–	–	–	–
Glutaredoxin-C3	2	–	–	–	2	2
Helicase	–	–	–	–	1	1
Metallophos_2 containing protein	2	–	–	–	–	–
MORN repeat-containing protein	3	8	2	2	4	4
mRNA-decapping protein g5R	–	1	–	–	–	–
Patatin	–	–	–	–	1	1
Putative ATP-dependent RNA helicase L377	–	1	–	–	–	–
Putative ATP-dependent RNA helicase R563	–	1	–	–	–	–
Putative DNA polymerase family X	–	–	1	1	–	–
Putative DNA-directed RNA polymerase subunit D	–	1	–	–	–	–
Putative helicase C962R	–	–	2	2	2	2
Putative histidinol-phosphate aminotransferase	–	–	–	–	1	1
Putative hydrolase	–	–	1	1	–	–
Putative phosphatidylglycerophosphate synthase	–	–	1	1	–	–
Putative poly(A) polymerase catalytic subunit	–	–	–	–	1	1
Putative T4-like proximal tail fiber			1	1	–	–
Putative UV-damage endonuclease			2	2	–	–
Ribonucleoside-diphosphate reductase large subunit	1	–	2	2	2	2
Ribonucleoside-diphosphate reductase small chain			1	1	–	–
RNA polymerase II subunit Rpb5b	–	1	–	–	–	–
Subunit common to RNA polymerases I, II, and III	1	–	2	2	3	3
Translation initiation factor SUI1	–	1	–	–	–	–
Transcription factor S-II-related protein	–	–	–	–	1	1

**Figure 2:**
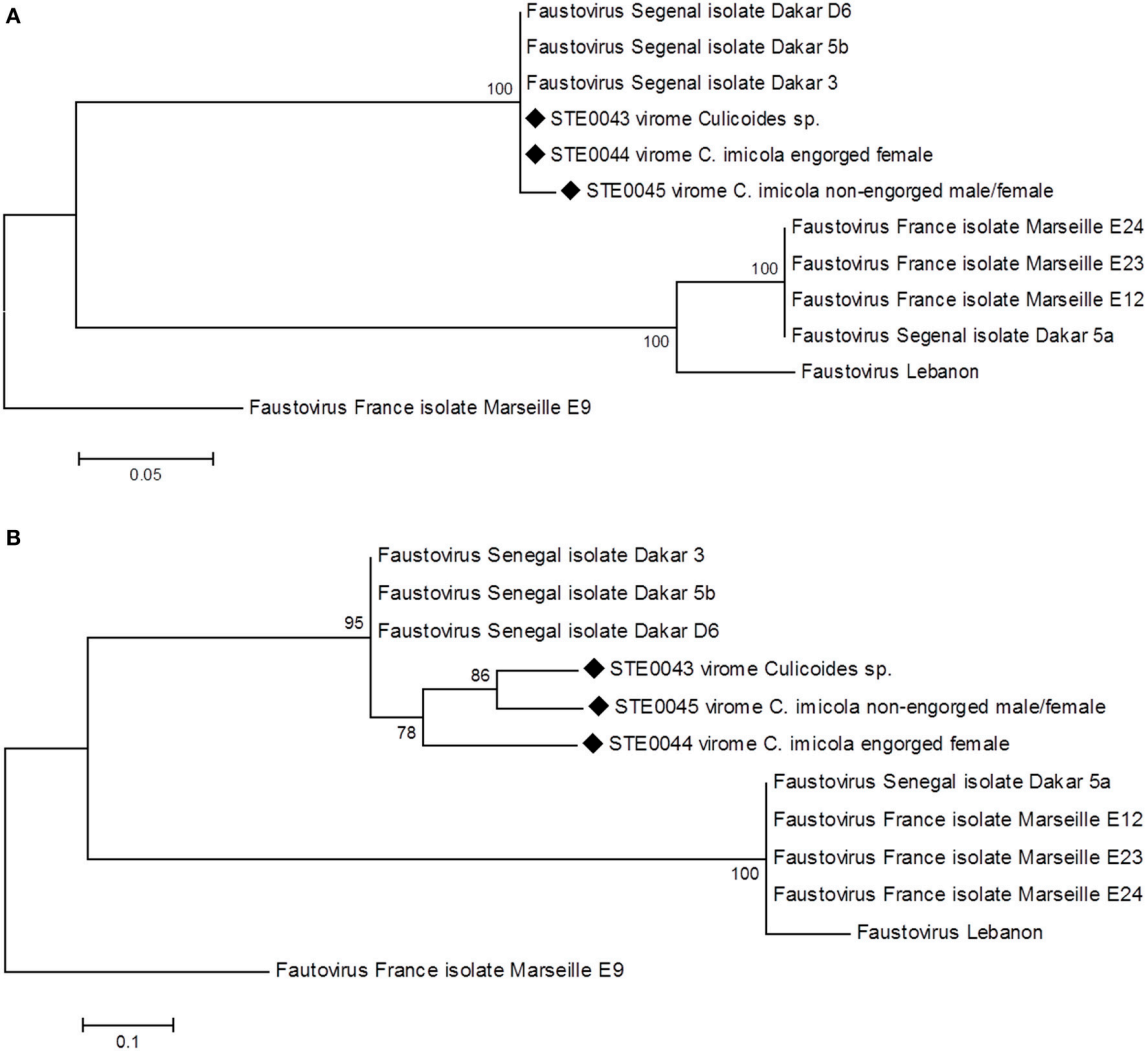
**Phylogenetic reconstruction of Faustovirus detected in biting midges' viromes based on the (A) nucleotide sequence of the sub-unit common to RNA polymerase I–II–III (substitution model: Kimura-2-parameters) (B) amino-acid sequence of the RNA diphosphate reductase large sub-unit (substitution model: JTT+G, *G* = 3)**.

Phylogenetic analyses performed on the sub-unit common to RNA polymerase I–II–III (Figure 2A) and on the RNA diphosphate reductase large sub-unit genes (Figure 2B) showed that the biting midges' Faustovirus grouped with other Faustoviruses isolated from French, Senegalese and Lebanese sewage. More precisely, Senegalese biting midges' Faustovirus formed a cluster with Dakar sewage Faustovirus, supported by high bootstrap nodes (100 and 95, respectively). These results were confirmed with phylogenetic analyses performed on the DNA topoisomerase (Supplemental Figure 2A) and the putative helicase C962R (Supplemental Figure 2B) genes.

Phylogeny performed on the RNA diphosphate reductase large sub-unit gene allowed distinguishing a specific cluster composed of biting midges Faustovirus only within the Senegalese Faustovirus clade, with a high bootstrap value of 78 (Figure 2B).

### Observation of virus-like particles by transmission electron-microscopy and isolation of a Faustovirus-like virus from biting midges

**Figure 3:**
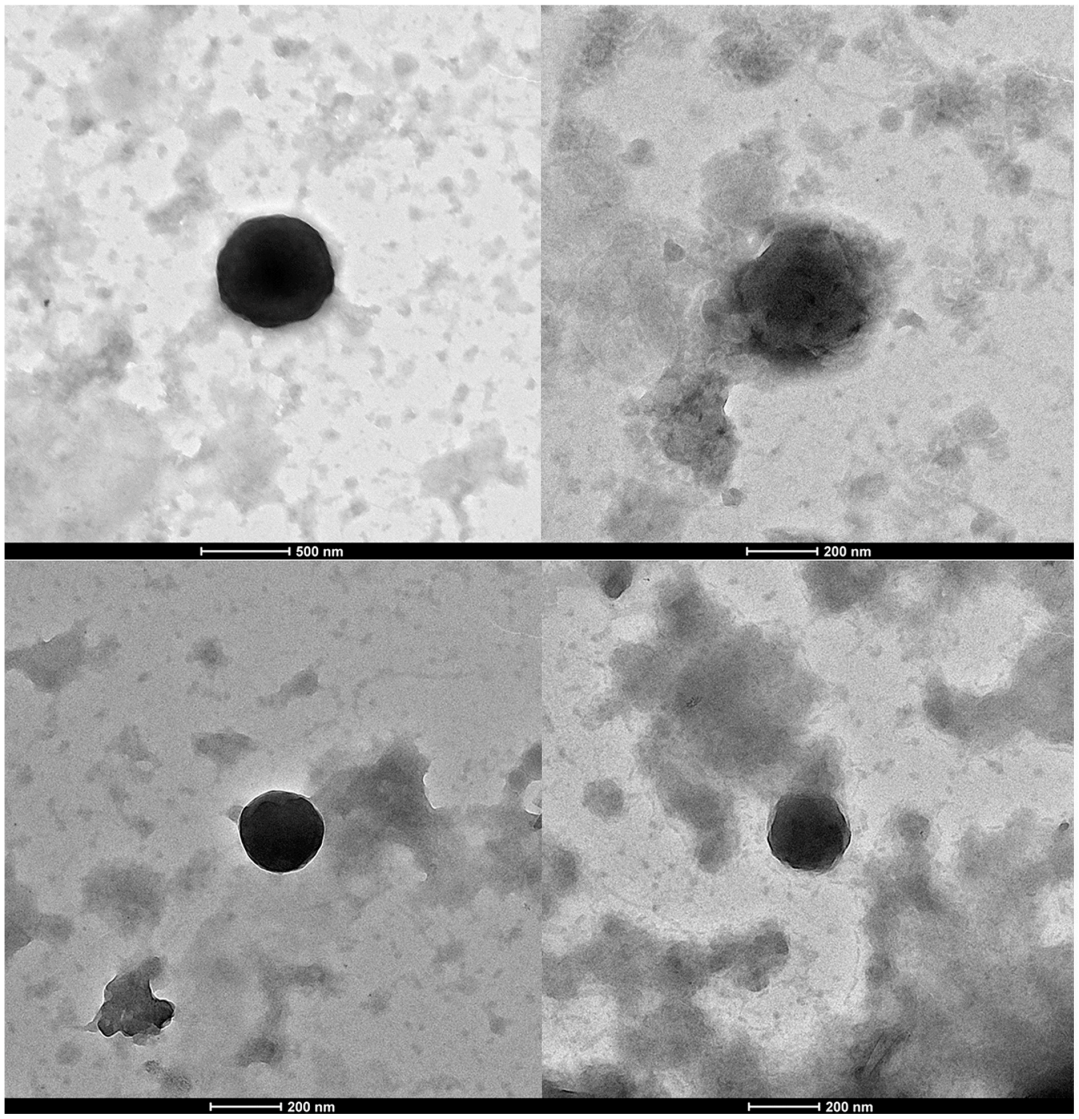
**Negative staining electron microscopy imaging of viral particles from *Culicoides* sp. biting midges' samples**. Scale bars are indicated under the images.

Viral particles purified from biting midges were negatively stained and observed by transmission electron microscopy (Figure 3). Virus-like particles were observed with different morphologies and diameters, ranging from 600 (Figure 3A) to 200 nm (Figure 3D). Some of the observed virions had a diameter (approximately 200 nm) and morphology (icosahedral capsid) compatible with that of Faustovirus (Figure 3D).

*V. vermiformis* protists was used in an attempt to isolate Faustovirus-related viruses detected in STE0043 *Culicoides* sp. virome. One *V. vermiformis* sub-culture was Faustovirus PCR-positive at Day 3 post-infection, and sequencing of a fraction of the capsid gene confirmed that the isolated virus was Faustovirus. However, successful viral production was impaired due to the high bacterial load present in the culture, originating from the arthropods' guts.

### Global proteomics of biting midges

**Figure 4:**
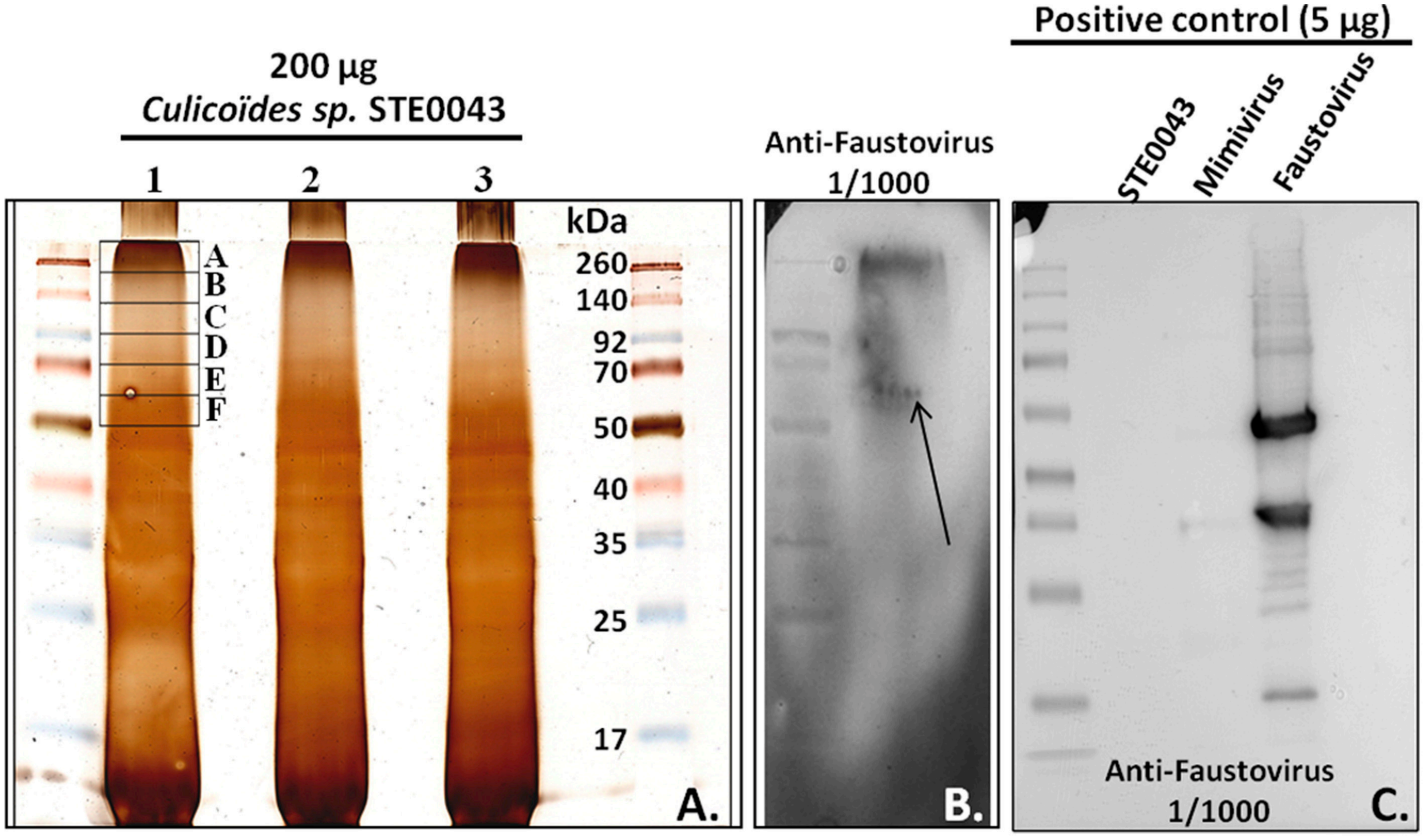
**Western blot analysis. (A)** Silver staining. **(B)** Staining with anti-Faustovirus polyclonal antibodies on arthropods sample. **(C)** Positive control of Faustovirus western blot with a loading of 5 μg of proteins.

Western blot analysis of STE0043 proteins revealed an immunoreactive smear, between 260 and 50 kDa, with anti-Faustovirus polyclonal antibodies (Figure 4B). The smear was due to a high load of proteins (200 μg) allowing the detection of viral proteins in very low abundance in these arthropods. As shown in Figure 4C, with a loading of 5 μg, no detection was possible in arthropods, although the positive control revealed reactive bands. Within this smear, a putative band at 60 kDa was observed for Faustovirus, possibly corresponding to the viral capsid (arrow of Figure 4B). Six pieces of electrophoresis-fractioned proteins among the immunoreactive smear were subsequently extracted from the gel and analyzed by proteomics (Figure 4A).

The global proteomic analysis of the six immunoreactive bands identified 4576 different peptides. Many peptides were not identified, due to incomplete databases. The major identified proteins were related to blood tissues: indeed, nearly a quarter of the total identified peptides represented blood proteins components, i.e., serum albumin, hemoglobin and fibrinogen, which represented 5.44, 15.57, and 0.90% of the total identified peptides, respectively. Numerous arthropod-borne peptides (33.77%) were also identified, and more than two thirds of identified peptides were related to the arthropods' major blood meal hosts: *Bovidae* represented 25.00% of the total identified peptides, 15.77% for humans, and 11.42% for *Rodentia*. Less abundantly, 2.95% of peptides were identified as coming from horses, 2.80% from pets (cats and dogs), 1.99% from *Lagomorpha* (mainly rabbits), 1.17% from swine, 1.05% from primates, and 0.87% from *Chiropera*. Interestingly and although no peptide matching with Faustovirus was detected, three peptides matched with the viral proteome of Saudian virus, a new giant virus isolated from sewage, were detected.

Similar results were obtained for the whole proteomic analysis, with slight differences in the proportions of identified peptides, except that four and seven peptides were obtained for Kroon virus and Courdo11 *Mimiviridae* proteins, respectively (Yoosuf et al., 2014; Boratto et al., 2015). In addition, *Mimiviridae*-related sequences were obtained in the metagenomes (data not shown). No match with Faustovirus was obtained.

No hit were obtained when comparing the peptides to the virome-predicted ORFs database.

### Detection of Faustovirus in animals and environmental water

The serum from 14 cattle and their associated hard ticks, 13 rodents (four suckling mice and nine *Arvicanthis* sp.), well water from Dielmo and Ndiop, and Néma river water recovered in Dielmo were collected and used to screen for the presence of Faustovirus.

The presence of Faustovirus was detected in five over 13 rodents tested: two were from suckling mice trapped in Ndiop and three were from *Arvicanthis* sp. rodents trapped in Ndiop (*N* = 2) and Dielmo (*N* = 1) respectively. Additionally, Faustovirus was detected in two over 14 cattle sera (one from Ndiop on a healthy cattle and one from Dielmo on a lumpy skin disease-infected veal). Faustovirus was also present in environmental water in both well and river waters from Dielmo and well water from Ndiop.

Two pools of hard ticks (*Boophilus* sp. and *Rhipicephalus evertsi*) collected from the same cattle in Keur Samba Gueye, a village close to Ndiop, were also positive for Faustovirus although the animal serum was negative. One pool of *Amblyomma* sp. hard ticks, collected from cattle in Ndiop, and one pool of *Ornithodoros sonrai* soft ticks, collected from rodents' nests in Keru Serigne Korka (a village located 12 km north-east of Dielmo) were also positive.

These positive detections were all confirmed by sequencing the portion of the DNA polymerase and the capsid genes of Faustovirus. Phylogenetic analyses of rodent-borne, cattle-borne, and water-borne Faustovirus performed on the capsid gene confirmed the relatedness of environmental and mammalian Faustovirus with arthropod-borne Faustovirus, but was not sufficiently discriminant to specifically define clusters of Senegalese Faustovirus (data not shown).

**Figure 5:**
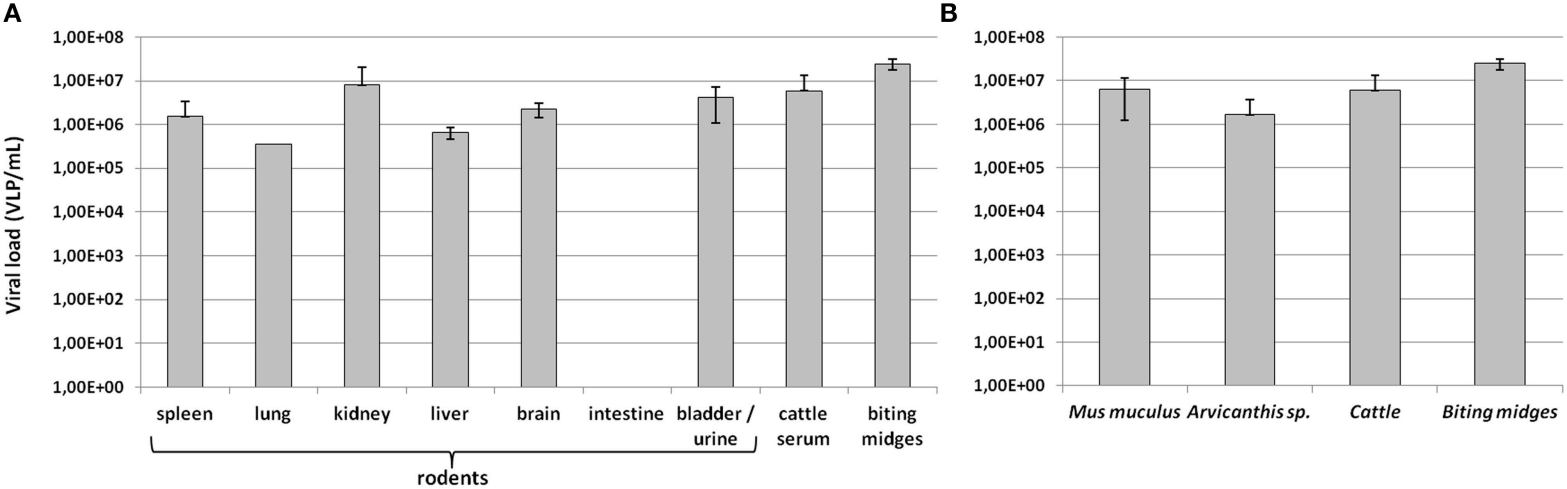
**Quantification of Faustovirus viral loads according to (A) tissue samples (B) animal species**. Viral loads are expressed in log_10_ VLP/mL.

Viral loads of Faustovirus in PCR-positive animals were estimated according to the tissue sample (Figure 5A) and the animal species (Figure 5B). All harvested organs were positive for Faustovirus, in viral loads ranging from 3.49 × 10^5^ VLP/mL (lung) to 8.01 × 10^6^ VLP/mL (kidney), except for intestine samples which were all negative, even when the extracted DNA was diluted in case of the presence of inhibitors. Interestingly, Faustovirus quantification in kidneys was similar in scale to that in the bladder or urine samples (4.21 × 10^6^ VLP/mL). Faustovirus load in cattle sera was estimated at 5.96 × 10^6^ VLP/mL. Biting midges non-amplified viromes were detected with the highest viral loads, estimated at 2.47 × 10^7^ VLP/mL (Figure 5A). No major difference was observed when analyzing the relative abundance of Faustovirus according to animal species (Figure 5B). Arvicanthis-positive animals presented the lowest (1.67 × 10^6^ VLP/mL) and biting midges the highest (2.47 × 10^7^ VLP/mL) Faustovirus load.

### Detection of Faustovirus in humans

The serum of 112 febrile patients with no known etiology and 51 healthy people was screened for the presence of Faustovirus with the DNA polymerase targeted system. A total of 11 out of 112 (9.82%) febrile patients and six out of 51 (11.76%) healthy persons were positive. Sequencing of the small PCR product (99 bp) confirmed the positive detection of Faustovirus on 57 bp. Capsid and DNA topoisomerase amplifications of Faustovirus-positive human sera were negative and RNA polymerase amplification of Faustovirus-positive human sera resulted in a non-specific multi-band amplification (data not shown).

## Discussion

Amoeba-infecting giant viruses have been isolated in a wide variety of biomes, mostly in environmental (i.e., soil and water) samples (Pagnier et al., 2013). Various amoeba-infecting giant viruses have also been detected in animals, such as in arthropod larvae (Boughalmi et al., 2013a), in the leech *Hirudo medicinalis* (Boughalmi et al., 2013b) or in the sera of cattle and monkeys (Dornas et al., 2014). Recently, the first amoeba-infecting giant virus belonging to the *Asfarviridae* family, Faustovirus, was reported in sewage in various geographical locations (Reteno et al., 2015). The unique other member of the *Asfarviridae* family is the ASFV, a tick-borne virus. In this study we detected and isolated for the first time Faustovirus in adults biting midges and their blood meal-associated mammals.

As part of a global study of viral communities existing in biting midges, pools of *Culicoides* sp., engorged female *C. imicola* and non-engorged male and female *C. imicola* were collected and their corresponding DNA viromes were sequenced. Results revealed the presence of sequences related to giant viruses, mainly Faustovirus with more than 90, 40, and 74% of total giant viral reads, respectively. The presence of an amoeba-infecting *Asfarviridae*-like virus in adult biting midges leads us to question on the mode of contamination of adult biting midges. *Ceratopogonidae* are arthropods with an aquatic and semi-aquatic larval stage, leading to possible contact with amoebae and their associated giant viruses during the larval stage, and a putative trans-stadial transmission of free viral particles or infected amoebae. Moreover, Evans and Schwarz (2011) reported the infection of adult honeybees by *Malpighamoeba mellificae*, a protozoan developing in the Malpighi tubes of honeybees. We can therefore hypothesize that, adult biting midges could either be infected at the larval or during the adult stage, either with free viral particles or infected amoebae. The mode of contamination of adult biting midges is currently unknown but further studies regarding the presence of Faustovirus in all stages of development of arthropods may help to solve this question.

The engorged female *C. imicola* virome showed the highest abundance of sequences related to Faustovirus, with significant differences with the non-engorged metagenome, suggesting a possible additional viral load of the arthropod via the blood meal of female biting midges. We then searched for the presence of sequences related to Faustovirus and other giant viruses in publicly available arthropods metagenomic datasets. Our results showed that, although in low abundance, giant viral contigs were detected in other arthropods, except for Faustovirus, never detected elsewhere than in the biting midges virome. Mosquitoes and body lice presented similar abundances of *Mimiviridae*- and *Pandoraviridae*-related contigs and hard ticks presented similar abundance of *Mimiviridae*-related contigs, whereas termites and whiteflies present no giant viral sequence. One should note that mosquitoes, body lice and hard ticks are hematophagous arthropods whereas termites and whiteflies are non-hematophagous arthropods, suggesting again the putative role of blood meal in the presence of giant viruses in adult arthropods. Further proteomic analysis of the pool of *Culicoides* sp. revealed the presence of *Bovidae, Rodentia*, and human blood-related proteins. As a consequence we subsequently screened for the presence of Faustovirus in human sera, cattle sera and rodent organs, and detected five Faustovirus-positive rodents and two Faustovirus-positive cattle, confirming the possible contamination of female biting midges via their blood meal. Additionally, we detected three Faustovirus-positive cattle-associated engorged hard ticks, again confirming possible infection of arthropods via their blood meal.

Interestingly, we reported high levels of Faustovirus either in rodent tissue or cattle sera (Figure 5), and the highest viral loads were found in rodents' kidney and urine samples, suggesting a possible excretion of Faustovirus by rodents in the environment. Finally, the Néma River and the well water from Dielmo and Ndiop were all positive for Faustovirus, suggesting a possible source of contamination of humans and animals via recreational or drinking water. In sub-Saharan countries, such as Senegal, biting midges usually feed on livestock but also on humans, resulting in the vector-borne transmission of pathogens to animals and humans. In this study we reported the detection of Faustovirus in human sera harvested from febrile patients and from healthy people, with no significant difference in the prevalence between the two groups. Although we could not conclude on a putative pathogenic role of Faustovirus, the questions of the mode of infection to humans have to be addressed: is Faustovirus vector-transmitted? And if so, what kind of arthropod can transmit the virus? Or do humans acquire Faustovirus via an environmental source (water, urines of rodents, etc)? The possible reservoir role of rodents in the viral cycle of Faustovirus, both in humans and arthropods, requires further investigations, as for the vector competence of arthropods for Faustovirus.

Faustovirus-related sequences were the most abundant in all viromes. Although no capsid sequence was detected in the metagenomes, we successfully amplified a fragment of the capsid gene and confirmed by sequencing (Supplemental Figure 1). Western blot analysis of *Culicoides* sp. proteins using Faustovirus antibodies highlighted a band at the expected size of the capsid protein (Figure 4B), and further mass spectrometry sequencing identified giant viral peptides, although not related to Faustovirus. Additionally, the observation of viral particles by transmission electron microscopy with a size and shape compatible with Faustovirus, and further successful isolation of Faustovirus conducted on *V. vermiformis*, a protist commonly found in human environments (Nazar et al., 2012; Coşkun et al., 2013; Niyyati et al., 2014), confirmed the presence of infectious viral particles in the *Culicoides* sp. pool of biting midges. Phylogenetic analyses performed on several core genes revealed that Faustovirus-like viruses detected in the three biting midge viromes branch together in a cluster formed by Dakar 5b, Dakar 3, and Dakar D6 Faustovirus (Figure 2), viruses that were previously isolated in sewage from Dakar, Senegal (Reteno et al., 2015). According to the phylogenetic analysis conducted on the capsid gene, rodent-borne, cattle-borne and water-borne Faustovirus clustered together with arthropod-borne Faustovirus. Unfortunately, this portion of the genome of Faustovirus was not sufficiently discriminant to be able to refine the classification of mammalian, arthropod and environmental Faustovirus. Complete full genome sequencing and characterization of these viruses will enable the phylogenetic relationships between arthropod-associated Faustovirus, environmental/mammalian Faustoviruses and human Faustovirus to be refined.

Faustovirus is a recently described giant virus infecting *V. vermiformis* amoebae (Reteno et al., 2015), whose close relative is the ASFV, the only member of the *Asfarviridae* family. *Asfarviridae* are tick-borne dsDNA viruses transmitted by *Ornithodoros* sp. soft ticks and responsible for the African swine fever, a highly contagious and fatal pig infection (Burrage, 2013; Hubálek et al., 2014). Recently, hard ticks have been suspected to be capable of transmitting the virus but without success, although viral DNA was detected up to 8 weeks post-inoculation (de Carvalho Ferreira et al., 2014). In our study we report the detection and isolation of an *Asfarviridae*-like Faustovirus in biting midges, but also in a pool of *O. sonrai* soft ticks and in *Boophilus* sp., *R. evertsi*, and *Amblyomma* sp. pools of hard ticks. Interestingly, *O. sonrai* soft ticks were collected in the dust contained in rodents' nests and were not engorged, whereas hard ticks were collected directly from livestock and were engorged. Additionally, nearly 40% of the rodents tested were Faustovirus-positive, suggesting that *O. sonrai* soft ticks could be a possible vector for Faustovirus, and rodents could be a putative reservoir since no symptoms were observed on the captured rodents and high loads of Faustovirus were detected in their kidneys and urine, resulting in possible excretion of the virus in the environment. The detection of Faustovirus in *Boophilus* sp., *R. evertsi*, and *Amblyomma* sp. hard ticks could reflect the blood meal of the ticks and the viral persistence of the virus or the viral DNA within the arthropod, as previously reported (de Carvalho Ferreira et al., 2014).

So far, Faustovirus has been only detected in sewage in Marseille, Dakar and in Lebanon and Saudi Arabia (Reteno et al., 2015). In this study, we report for the first time the detection and isolation of Faustovirus in adult biting midges, and the detection of high viral loads of Faustovirus in rodents and cattle. We also reported the detection of Faustovirus in febrile patients and healthy people. This work thus, highlights the need to investigate the role of arthropods and wild or domestic animals on the lifecycle of the *Asfarviridae*-like Faustovirus and, more globally, for the amoeba-infecting giant viruses.

## Author contributions

ST, BL, OM, DR, CD designed the experiments. ST, SM, SA, PD, JBK, JB, PJ, CR performed the experiments. ST, MS, MA collected the samples. ST, SA, PD, JBK, JB, OM wrote the article. BL, OM, DR, CD revised the article.

## Funding

This work was conducted under the frame of the ANR-13-JSV6-0004 awarded to Christelle Desnues.

### Conflict of interest statement

The authors declare that the research was conducted in the absence of any commercial or financial relationships that could be construed as a potential conflict of interest.

## Supplementary material

Supplemental Figure 1 **Electrophoresis of PCR targeting the Faustovirus capsid conducted on biting midges viromes**.

Supplemental Figure 2 **Phylogenetic reconstruction of Faustovirus detected in biting midges viromes based on the (A) nucleotide sequence of the DNA topoisomerase small sub-unit (substitution model: HKY) (B) nucleotide sequence of the putative helicase C962R (substitution model: HKY)**.
