# Supplementary figures and images for: Faustovirus-Like Asfarvirus in Hematophagous Biting Midges and Their Vertebrate Hosts
Source: Front Microbiol. 2015 Dec 16;6:1406. doi: 10.3389/fmicb.2015.01406 (PMC4679923; doi:10.3389/fmicb.2015.01406)

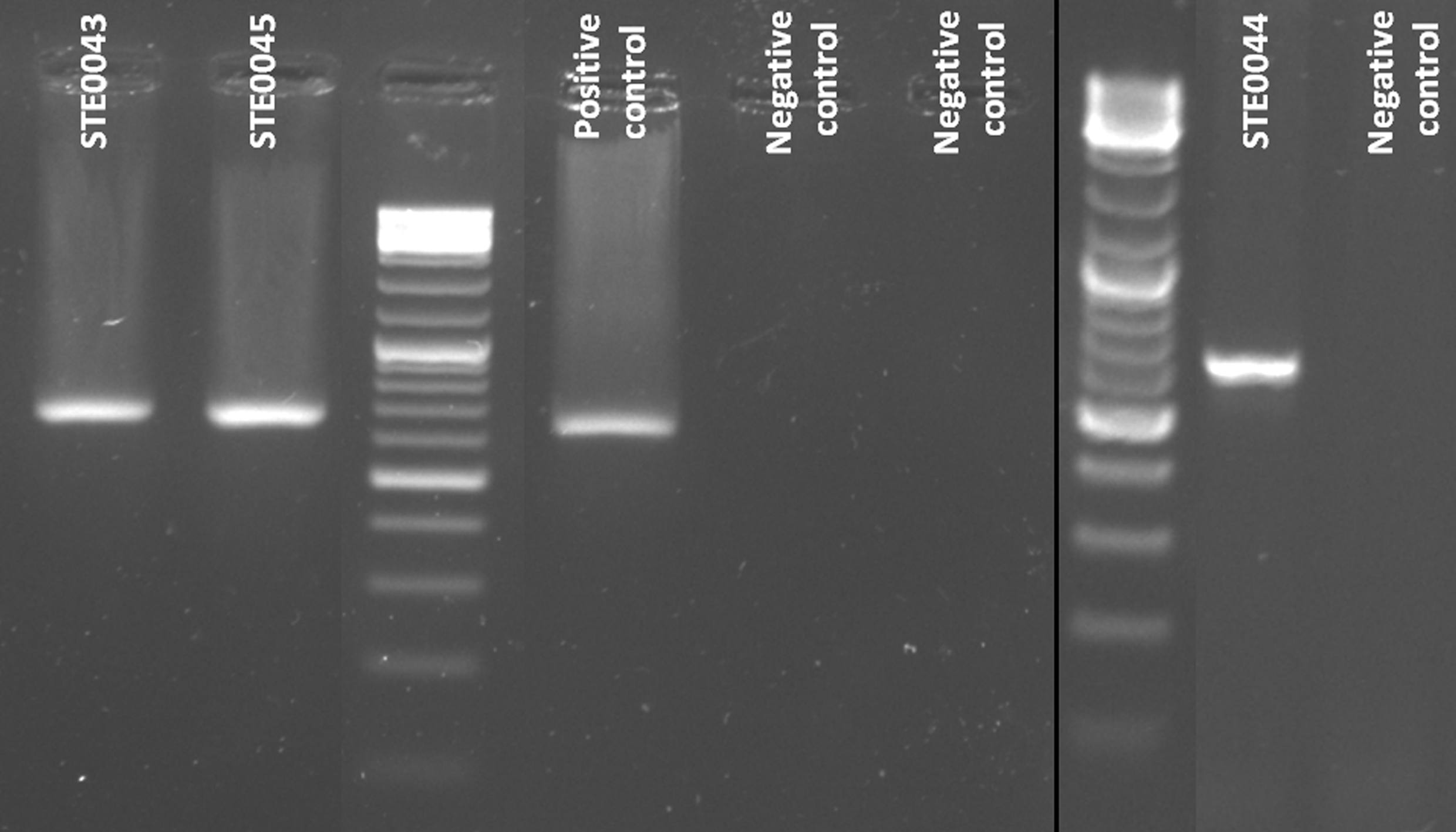

Supplement: Supplemental Figure 1 — Electrophoresis of PCR targeting the Faustovirus capsid conducted on biting midges viromes. [file Image1.TIF]

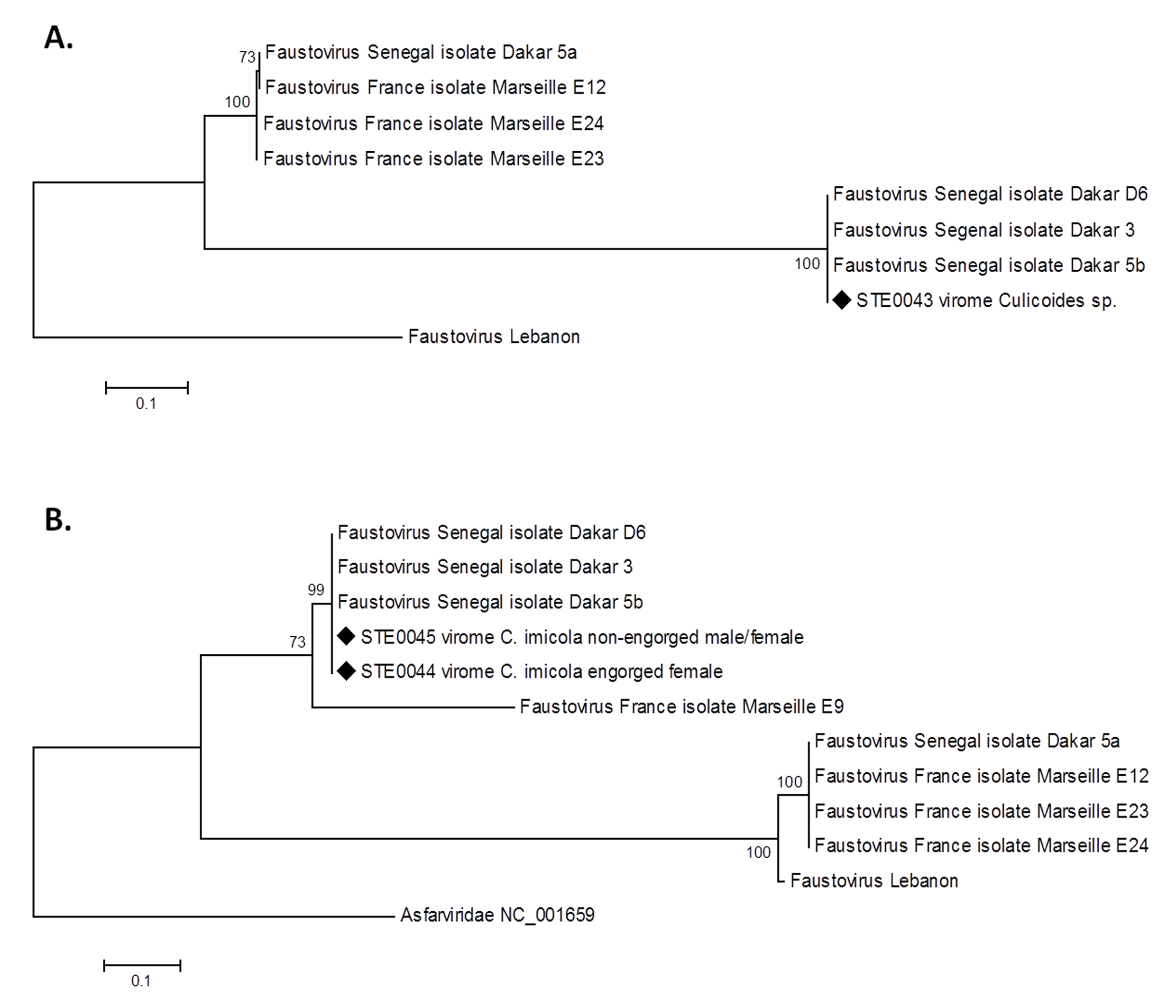

Supplement: Supplemental Figure 2 — Phylogenetic reconstruction of Faustovirus detected in biting midges viromes based on the (A) nucleotide sequence of the DNA topoisomerase small sub-unit (substitution model: HKY) (B) nucleotide sequence of the putative helicase C962R (substitution model: HKY). [file Image2.TIF]
